# Integration of *“omics”* Data and Phenotypic Data Within a Unified Extensible Multimodal Framework

**DOI:** 10.3389/fninf.2018.00091

**Published:** 2018-12-18

**Authors:** Samir Das, Xavier Lecours Boucher, Christine Rogers, Carolina Makowski, François Chouinard-Decorte, Kathleen Oros Klein, Natacha Beck, Pierre Rioux, Shawn T. Brown, Zia Mohaddes, Cole Zweber, Victoria Foing, Marie Forest, Kieran J. O’Donnell, Joanne Clark, Michael J. Meaney, Celia M. T. Greenwood, Alan C. Evans

**Affiliations:** ^1^McGill Centre for Integrative Neuroscience, Montreal Neurological Institute, Montreal, QC, Canada; ^2^Montreal Neurological Institute, McGill University, Montreal, QC, Canada; ^3^Douglas Hospital Research Centre, McGill University, Montreal, QC, Canada; ^4^Ludmer Centre for Neuroinformatics & Mental Health, McGill University, Montreal, QC, Canada; ^5^Lady Davis Institute, Jewish General Hospital, McGill University, Montreal, QC, Canada

**Keywords:** workflow, omics analysis, integrative neuroscience, reproducibility, database, HPC, genomics, biostatistics

## Abstract

Analysis of “*omics*” data is often a long and segmented process, encompassing multiple stages from initial data collection to processing, quality control and visualization. The cross-modal nature of recent genomic analyses renders this process challenging to both automate and standardize; consequently, users often resort to manual interventions that compromise data reliability and reproducibility. This in turn can produce multiple versions of datasets across storage systems. As a result, scientists can lose significant time and resources trying to execute and monitor their analytical workflows and encounter difficulties sharing versioned data. In 2015, the Ludmer Centre for Neuroinformatics and Mental Health at McGill University brought together expertise from the Douglas Mental Health University Institute, the Lady Davis Institute and the Montreal Neurological Institute (MNI) to form a genetics/epigenetics working group. The objectives of this working group are to: (i) design an automated and seamless process for (epi)genetic data that consolidates heterogeneous datasets into the LORIS open-source data platform; (ii) streamline data analysis; (iii) integrate results with provenance information; and (iv) facilitate structured and versioned sharing of pipelines for optimized reproducibility using high-performance computing (HPC) environments via the CBRAIN processing portal. This article outlines the resulting *generalizable “omics” framework* and its benefits, specifically, the ability to: (i) integrate multiple types of biological and multi-modal datasets (imaging, clinical, demographics and behavioral); (ii) automate the process of launching analysis pipelines on HPC platforms; (iii) remove the bioinformatic barriers that are inherent to this process; (iv) ensure standardization and transparent sharing of processing pipelines to improve computational consistency; (v) store results in a queryable web interface; (vi) offer visualization tools to better view the data; and (vii) provide the mechanisms to ensure usability and reproducibility. This framework for workflows facilitates brain research discovery by reducing human error through automation of analysis pipelines and seamless linking of multimodal data, allowing investigators to focus on research instead of data handling.

## Introduction

Genomic analysis and bioinformatics have undergone a technological revolution over the past few decades, one that holds great promise for scientific discovery but also poses significant big-data challenges. To increase accessibility for researchers with varying levels of informatics expertise, the “Big Data” components of “*omics”^1^* analyses need to be integrated into an automated and seamless workflow. To this end, in 2015 the Ludmer Centre for Neuroinformatics and Mental Health^2^ created a genetic/epigenetic working group composed of three member institutions of McGill University: (i) the Douglas Mental Health University Institute, focusing on biological questions; (ii) the Lady Davis Institute at the Jewish General Hospital, focusing on tools for statistical analysis; and (iii) the McGill Centre for Integrative Neuroscience at the Montreal Neurological Institute (MNI), responsible for the neuroinformatics infrastructure (Das et al., 2016, 2017).

The goal of the working group is the integration of “*omics*” data into the LORIS data platform^3^, a web-based open-source data and project management platform (Das et al., 2011) to streamline analysis, integrate results, and facilitate structured sharing for optimized reproducibility, using high-performance-computing (HPC) environments via CBRAIN^4^ (Sherif et al., 2014), a web-based open-source platform that allows computationally intensive analyses of data by connecting researchers to HPC facilities. The pilot use-case for multimodal “omics” workflow integration focused on analysis outputs from the *Methylation450k*^5^ pipeline, a functional normalization pipeline for epigenomic data from a Ludmer Centre-based study.

This article describes an extensible and adaptable framework that addresses critical gaps in integrating “omics” data with multi-modal phenotypic datasets (imaging, behavioral, clinical, demographic, …) using HPC and databases, while leveraging standardization and automation to provide GUI-based workflows for less technical researchers. Analysis of data, specifically genomic or imaging, can involve multiple parallel paths. These workflows typically begin with the processing of biological samples, followed by quality control and analysis using data-specific pipelines, and culminate in querying and visualization of summary data. The complexity of such analyses often requires a framework that can comprehensively integrate these steps across data modalities, an element that is currently lacking in many existing *“omics”* toolboxes and workflows (Kanwal et al., 2017).

In designing such a framework, it is also important to consider features that would simplify and strengthen effective data sharing mechanisms, especially as we enter the era of Open Science. The processing of raw data is often performed by third-party platforms, whereby the resulting files are processed using one or more bioinformatic pipelines by the host laboratories.

One of the inefficiencies of this model is that each processing step typically generates a new version of the dataset, which is often stored on a local workstation or distributed across multiple drives. As quality control and post-processing tasks remove aberrant values, additional versions can multiply across storage systems, but without having sufficient transparency in the options or environment parameters used in the execution to generate each version (Glatard et al., 2015). Not surprisingly, this also leads to ineffective data-sharing, whereby it becomes unclear which copies of the data contain the most comprehensive and accurate information, requiring researchers to sift through redundant data.

A few systems have been created, such as the *Galaxy* platform for genomic data (Afgan et al., 2016, 2018) to integrate biological data and streamline genetic analysis (Kanwal et al., 2017). Many software platforms exist for sharing workflows to capture and promote the execution of reproducible analyses, such as Jupyter notebooks^6^. While such models seek to increase reproducibility in computational biology, they do not prioritize cross-modal data integration. Importantly, the field would benefit from a structured workflow that links organized cross-sectional or longitudinal multimodal data (genetics, imaging, behavioral) with HPC platforms for analysis (Poldrack et al., 2017).

We have leveraged existing architectures to create a model that aims to abstract the complexities of multi-modal processing and analysis. This combined framework builds upon systems documented in previous publications (Das et al., 2016, 2017) and integrates additional technologies and feature-layers to support an approach that prioritizes the: (i) integration of heterogeneous biological data with multi-modal datasets (imaging, clinical, demographics and behavioral); (ii) automation in launching analysis pipelines on HPC platforms; (iii) removal of technical barriers that are inherent to this process (Pool and Esnayra, 2000); (iv) standardization and transparent sharing of processing pipelines to improve computational consistency; (v) storage of results into a queryable web interface; (vi) feature rich visualization tools; and (vii) provision of mechanisms to ensure usability and reproducibility. The result is a streamlined approach for cross-modal analysis (such as imaging genetics) that also promotes the FAIR principles (Findable, Accessible, Interoperable and Reproducible) for data sharing (Wilkinson et al., 2016). The framework presented in this article can be used by researchers interested in integrating “*omics”* data with other multimodal datasets, such as those utilized in behavioral and/or imaging genetics projects, and can be readily modified to accommodate the specific needs of other users and projects.

## Materials and Methods

The goal of this “*omics*” framework is to take individual processing and analysis tasks, including any manual steps that might already exist, and integrate them into a more automated model that leverages: (i) standardization and harmonization tools; (ii) HPC resources; and (iii) application programming interface (API) interoperability for automation between the existing platforms. In this section, we describe the components of software and platforms, and recent extensions, which together support workflows for processing and transferring “*omics”* data.

The complexities of cross-modal workflows in “*omics*” analyses is a significant challenge for researchers given that such workflows are difficult to automate and require regular user intervention, support and maintenance. Tool development and integration at iterative stages of development is time-consuming and mandates thorough testing to successfully build a workflow. To this end, identifying the labor-intensive steps (file transfers, versioning, user access, etc.) of a data processing workflow and automating them is an essential priority.

**Figure 1:**
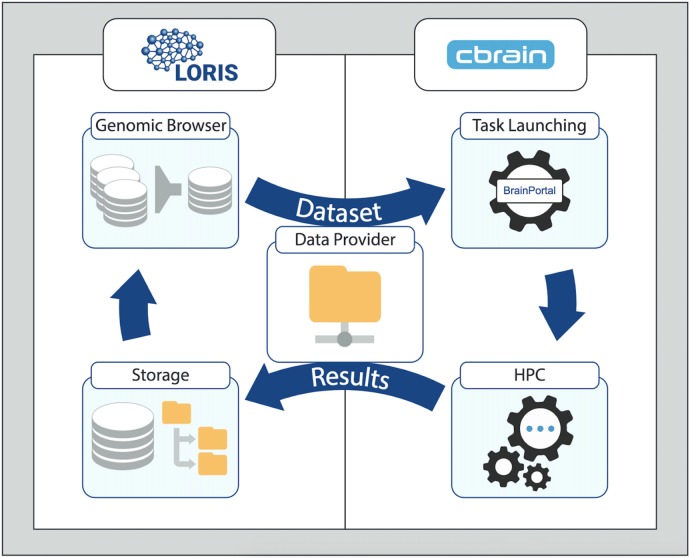
Generalized Workflow cycle between the LORIS data-management platform and the CBRAIN processing platform. Data from LORIS (Storage) can be queried and filtered (Genomic Browser and other tools) to select a set of variables and/or files. The newly created dataset is then transferred to the CBRAIN DataProvider for processing (Task Launching) and analysis (high-performance computing, HPC). The output is synced back to LORIS with the provenance data. Results can be examined and a new iteration can begin with the added derived variables. For stepwise details of this model, please see Figure 2 in “Results” section.

Building a generalized framework by extending the MNI ecosystem’s combined platform of LORIS and CBRAIN starts with populating the LORIS database with participant data for all modalities (such as behavioral, imaging and *“omics”*). For the two systems to communicate and exchange data as input or output of a given pipeline, a shared space must be defined. (This role can be served by a CBRAIN *DataProvider*, accessible to the LORIS filesystem). That is followed by the installation of tools on CBRAIN such that they can be launched on HPCs. Finally, customizations and extensions to LORIS can support new formats of data. Figure 1 shows the cyclical flow of data between LORIS and CBRAIN, whereby stored datasets are processed and their outputs returned as results.

A typical use-case begins with biological samples and phenotypic data collected during a subject’s visit. The biological/phenotypic samples are then processed on-site or shipped to a specialized facility for genomic analysis or image capture, after which raw data files are created and made available for statistical and/or bioinformatics analysis. Files containing raw data are stored in a LORIS database and then subsets are queried, selected and sent to CBRAIN to be processed by an analysis tool. The output is returned back to LORIS for storage along with its provenance metadata from the processing task. Summary and aggregate data can be parsed and explored through various LORIS modules and then queried to create new datasets linked to provenance metadata. This model allows for iterative processing as data selections can be resubmitted from LORIS for further processing and analysis tasks via CBRAIN, with derived results returned once again into LORIS for storage and dissemination. It should be noted that a specific use-case will be demonstrated in the Results section that focuses on genomic and epigenomic data; however, similar procedures would apply for other “*omics”* data types.

To illustrate this framework with a genomic processing workflow, the relevant components of the LORIS and CBRAIN platforms (and feature extensions) are described below. Also outlined are the structural design elements facilitated by RESTful^7^ API interoperability between the two systems including: (i) the data transfer mechanisms; (ii) the abstraction of data organization; and (iii) the pipeline execution flow. Key auxiliary components and technologies interfacing with these platforms are integral to the multimodal framework, including containerization of pipelines, visualization of genomic and epigenomic data and NoSQL data storage.

### LORIS Data Platform

The LORIS platform is the entry point for data in most workflows deployed on this integrated framework. LORIS can house data at various stages of the processing lifecycle, and can typically be customized with import pipelines to accept and validate files of any type. Imported files can then be parsed to extract and store any relevant values in relational database tables, which are accessed by web-facing front-end modules. For large files, the filesets themselves will be organized on the LORIS data partition, and linked by their file paths from individual database-table entries, which serve as pointers to the data location on the server. Metadata for these files can also be stored in database tables in a key-value pair format, which is also an extensible structure that accepts any data format. File paths and metadata are easily accessible via LORIS’ front-end modules, through which users can peruse, filter, visualize and retrieve these datasets for download or export to other systems via the user-friendly web interface. Later in under the “LORIS Genomic Browser” section, we expand upon new “*omics*” features in LORIS.

### CBRAIN

CBRAIN’s web-based portal for the Compute Canada^8^ network enables user-friendly deployment and execution of pipelines across the Canadian HPC grid. For LORIS to launch a data processing task^9^ through CBRAIN, the interface between these systems must define the expected types and formats for both inputs and outputs.

Several key CBRAIN features support the workflow model across platforms. First, data storage and transfers are handled by a *DataProvider* (a designated file server space which connects to CBRAIN and the HPC grid), which caches and tracks data files across the HPC network. Second, CBRAIN’s *ToolConfiguration* profile enables rapid setup and user-friendly re-use of a scientific tool, describing where and how it is available on the supercomputer clusters, as well as defining the cluster setup parameters (environment setup, CPUs used, queue name, etc.) and input parameters required for executing the tool.

The *ToolConfiguration* can be automatically generated in CBRAIN through a Boutiques descriptor (Glatard et al., 2018) which provides a standard JSON protocol for defining the command-line and input and output variables for pipeline execution. Typically, this initial setup needs to be configured only once, thereafter allowing for re-use of the same software setup by providing the proper input parameters. Together, the *DataProvider* and *ToolConfiguration* abstract the infrastructural complexities of data storage, transfer and processing parameters for the user while promoting transparency and reproducibility.

While CBRAIN supports the direct installation of pipelines for execution on HPC clusters, it has also introduced support for container technologies to specify the environment and package versions for optimally pre-defined execution of such pipelines.

#### LORIS DataProvider for CBRAIN

The *DataProvider* acts as a shared file system, such that CBRAIN and LORIS can interoperate with file-level read and write access of both the data and metadata. On the CBRAIN side, files are read from the LORIS *DataProvider* repository and made available to the HPC network. Once processing has been completed on the HPC grid, results from the pipeline execution on CBRAIN are written to the LORIS *DataProvider*, and subsequently recognized and imported back into the LORIS database and file system.

To make the file system interaction easier for LORIS’ web application, a dedicated directory on the LORIS server is designated as the *DataProvider*. Both CBRAIN and LORIS can read and write to this directory, which effectively allows for communicating datasets between platforms along with accompanying metadata.

#### Preparation of Pipelines (Containers)

To facilitate the flexible and reproducible integration and deployment of new tools across different HPC resources, CBRAIN and other execution platforms support containerization technology such as Docker^10^ and Singularity^11^. A container encapsulates the setup of the processing environment as well as any specific support packages that are needed, thereby making installation of software architecture independent, which improves reproducibility of analysis. Typically, an accompanying container description file^12^ describes every step necessary to construct the container. This provides the benefit of organizing and recording each aspect of the pipeline, and facilitates transparency in defining the runtime environment in a shareable, versionable document.

Additionally, by documenting the input parameters for the pipeline, specific aspects of the pipeline run can be adjusted and tracked in a controlled manner ensuring that all other factors stay the same, such as running the same pipeline using a different R package for functional normalization. For instance, the *Methylation450k* pipeline, which provides quality control (QC) and functional normalization of the Illumina 450k beadchip array data, currently integrates the *funNorm* (Fortin et al., 2014) R package. However, the flexibility offered by container-defined plug-ins and parameters enables a user to rapidly relaunch the same pipeline on a similar R package* funtooNorm* (Oros Klein et al., 2016), providing a clearly documented trace of provenance for comparison of results between the two normalization algorithms.

Another example is the *imputePrepSanger^13^* pipeline from the Ludmer Centre. This tool prepares PLINK genotype files to be sent to the Sanger Institute’s online Imputation Service^14^ by performing quality control, adjusting the positions and strand alignment of PLINK files, then converting them to VCF^15^ for submission to the Sanger server. The pipeline execution parameters were defined in a container on CBRAIN.

A third pipeline, *principal component of explained variance* (*PCEV*)^16^, was prepared to run a dimension-reduction algorithm to explain a maximum of variance in a response vector governed by a set of covariates. Specifically, this tool can be run multiple times, using different genomic-ranges to provide a new set of methylation Beta-values and genomic variants and/or a different set of covariates from behavioral and imaging metrics.

This model can be adapted for larger workflows, enabling reproducible execution of pipelines as a generalizable concept that could be applied to many use-cases. Examples include automatically running a piece of software when new data are available, performing quality control or validation, or ensuring that users run the same tool version in the same runtime conditions throughout the lifecycle of a study.

#### CBRAIN/LORIS Hooks

In order for data to pass seamlessly from one system to another, communication occurs between LORIS and CBRAIN using a RESTful (web) API for requests, and the *DataProvider* for data transfer and registration. A client for the CBRAIN API written in the PHP programming language has been created using SwaggerEditor^17^ with a schema^18^ following OpenAPI specification v2.0, which allows LORIS to look at available files and tools on CBRAIN. This PHP client also abstracts the handling of HTTP GET and POST requests which trigger the creation of new processing tasks on the HPC grid via CBRAIN. For a newly generated dataset, LORIS starts by registering the files in CBRAIN, making it possible to run relevant tasks. The type of the tasks, their parameters, and input files are then communicated through the API to CBRAIN, which launches them.

A LORIS process running in the background monitors a CBRAIN task’s status. The task progress can be followed from LORIS’ Server Processes Manager module. Capture of logs from data insertion and the task’s output from CBRAIN, as well as queries used to generate the new dataset, will be stored in a header file or in the database. This way, at the time of publication, all information describing provenance can be formatted in a file compliant with the Neuroimaging Data Model (NIDM; Keator et al., 2016).

### LORIS Genomic Browser

The *Genomic Browser* module (Rogers et al., 2015) is the principal LORIS component for visualization, querying, validation and storage of genomic and genetic data, and is part of an open-source feature set available on GitHub. This module enables browsing of single-nucleotide polymorphisms (SNPs) and copy number variants (CNVs) data, but has been expanded for this application to allow exploration of epigenomic data using the same functionalities. Any filtered subset of data can be downloaded and exported for further analysis, in addition to being passed to the visualization utilities embedded within the module. This allows for a genomic dataset to be viewed alongside behavioral and imaging data. The system includes functionality for viewing, filtering and linking of summary genetic data [CNV, SNP and other results from genome wide association studies (GWAS)]. Links to reference databases (UCSC genome browser^19^, dbSNP) have also been added.

#### Genomic Uploader

Genomic data is loaded into LORIS from raw or processed files using the web interface in the *Genomic Uploader*. This rudimentary upload tool is provided to facilitate loading and linkage of data files and records in the database. In addition to maintaining a reference for uploaded files, the uploader creates relations between inserted values, their annotations, and the study subject they belong to within the file header. When the file type fits a study’s expected types, user-defined scripts tailored to the genotyping platform of interest are provided. Inserted data are accessible and browsable in the module’s tabs.

#### Profile Summary Tab

The first tab of the *Genomic Browser* is called the Profile Summary tab and provides researchers with a high-level understanding of the data types available for individual subjects as well as summary statistics. This tab displays a sortable view of this information and enables filtering by population of interest and subject metadata for available genomic datasets stored in LORIS. The number of CNVs and SNPs or methylation CpGs found for each subject can be reviewed, filtered and sorted at a glance. By applying filters based on cohort or phenotypic gender, users can view these summary statistics for a sub-population of interest.

#### Genomic Browser Tabs: CNV, SNP, Methylation

Other tabs of the *Genomic Browser* provide subject-specific results for each data type from various epi-genomic and -genetic analyses (e.g., for CNV, SNP, or methylation results). When pipeline outputs are imported into LORIS and matched with an expected file format, the appropriate tab is automatically populated with data that is visible to the user. Each tab enables filtering by specific genomic regions around genes of interest or shared properties.

#### Genomic Viewer

An additional tab within the *Genomic Browser* was added to provide advanced exploration for epigenomic data, with genomic data aligning these points along the genome in superimposed tracks. This visualization technique is found in many domain-specific softwares and was developed for LORIS using *React.js*^20^ components for each track to dynamically render as page elements. Interactive display features are also created using *D3.js*^21^ visualization libraries for HTML5 canvas and SVG image generation. These combined technology layers can efficiently manage large volumes of data.

In our example implementation, the *Methylation450k* normalization pipeline produced a single output file containing Beta-values for all samples across all probes which were uploaded as a batch into LORIS via CBRAIN. Upon loading Beta-values^22^ into LORIS, each probe must be associated with an annotation record provided by the manufacturer of the array (Illumina). These annotation records are stored in the *genomic_cpg_annotation* database table which is populated using a script^23^ provided in the LORIS codebase. Each probe is then linked to a sample ID and a corresponding subject in the database. A mapping file is used in this process to link each sample to the subject ID.

The MySQL database contains paths to the three files (Figure 3) that comprise the dataset: the Beta-values file, the sample mapping file, and an annotation file. Once registered in the database, any type of biological data can be linked to behavioral and imaging data for each subject using their subject ID. The relationship between subjects and their biological data records is defined at the sample level, allowing for metrics from duplicate biosamples to be linked to the same subject. Once this link has been established, visualization tools within the *Genomic Browser* are used to look at available data for regions of interest on the genome. The SNP and CpG locations are aligned with histone marks or CpG islands, providing additional information about genomic features and regulatory interactions in the same locus.

**Figure 2:**
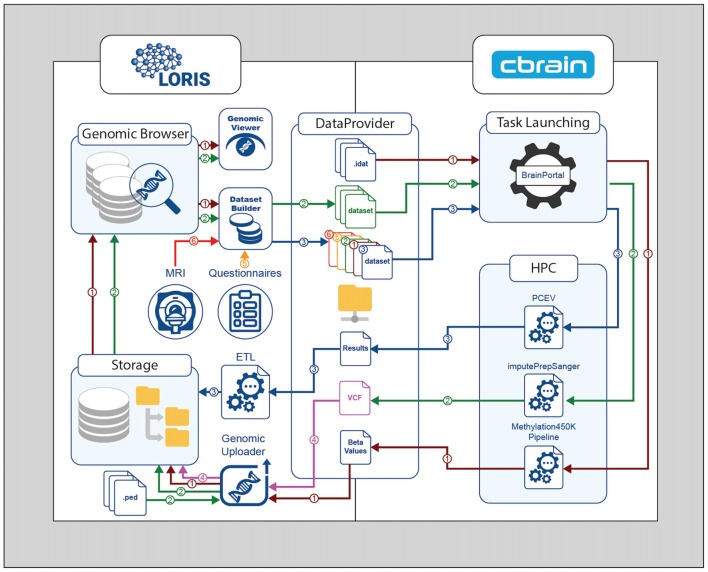
Genomic processing cycle between LORIS and CBRAIN through the DataProvider. Methylation450K pipeline—Brown path (1): IDAT files are transferred to the DataProvider, then the methylation normalization pipeline is launched. The Beta-values output file is returned to the DataProvider, and then loaded into LORIS using the Genomic Uploader. The inserted results can be browsed or visualized in the Genomic Browser module. ImputePrepSanger pipeline—Green path (2): PLINK files are added to LORIS via the Genomic Uploader, selected in the DatasetBuilder, and sent to CBRAIN for the imputePrepSanger tool to be run. The resulting Variant Call Format (VCF) output file is stored in LORIS—Pink path (4). Statistical analysis—Blue path (3): using the DatasetBuilder module in LORIS, data from any source (Orange path (5), Red path (6)) can be packaged in a new dataset and sent to CBRAIN via the DataProvider for statistical analysis using (e.g.,) the principal component of explained variance (PCEV) pipeline.

**Figure 3:**
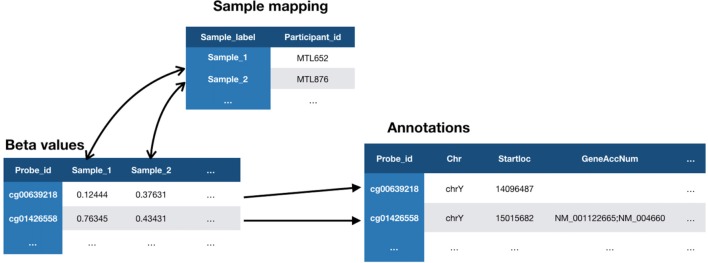
Relationship between three files required for loading of methylation data in LORIS’ Genomic Browser. The Beta-values file contains a value for each biosample tested on each probe. Each biosample in the Beta-values file is linked to a study subject in the Sample mapping file, using a subject identifier (Participant_id). Each probe from the Beta-values file is linked to a set of properties in the Annotations file provided by the chip manufacturer (Illumina).

#### Building Cross-Modal Queries

Within LORIS, a prototype *DatasetBuilder* module allows users to create new datasets by joining filtered genomic data with phenotypic data and/or imaging files queried from the Data Querying Tool (DQT; MacFarlane et al., 2014), to rapidly handle large datasets on the scale of genomic results, and provide that data to the user-facing frontend.

Both the DQT and the *DatasetBuilder* are built upon CouchDB, a file-based NoSQL database that provides a REST API for querying and filtering prebuilt data views. The views are generated by applying MapReduce^24^ algorithms, where each document is transformed using a mapping function and then summarized by the reducer function to create an indexed set of key-value pairs.

The *DatasetBuilder* processes an HTTP request issued for a specified *genomic_range* or DNA chip probe identifier, and retrieves all data records corresponding to the indexed range. For each record returned, a filter function identifies the samples of interest and extracts the Beta-values for display in the module. The subject IDs corresponding to these records are identified and a request is made to run an existing query saved in the DQT to select other phenotypic variables of interest (e.g., demographics, behavioral measures, etc.). The phenotypic datasets returned by the DQT are then joined with the biosample subject data to produce a combined dataset of fields across all modalities. These results are exported as CSV files to the CBRAIN *DataProvider* for further processing.

## Results

To demonstrate this framework for “omics” workflows, a specific “use-case” implementation from the Ludmer Centre working group is discussed, which includes genotyping, methylation assessments and typical phenotypic data (age, sex, etc.). The data was collected and derived from human subjects participating in a longitudinal study conducted by Ludmer researchers at the Douglas Mental Health University Institute in Montreal. The *Methylation450k* pipeline was run on the study dataset, and the outputs transferred via CBRAIN to LORIS. Using the *Genomic Browser* in LORIS, users could then query, select visualize and download data across phenotypic and epigenomic datasets. Further containers were created for additional pipelines such as *PCEV*, and installed and launched on the HPC grid via CBRAIN. The output of each task is transferred to the DataProvider and can then be loaded in the database, where it is linked to the provenance history of the task parameters and inputs.

Throughout this example, end-users seeking to reproduce, review, and use the data and metadata have the ability to use this complex pipeline with little technical knowledge through transparently accessible computing, negating the need to focus on: (i) transferring files across servers and clusters; (ii) managing versions; (iii) controlling user access; (iv) connecting with HPC units; (v) launching tasks; (vi) tracking progress; and (vii) capturing processing status, parameters and results. Once the outputs are stored and accessible in the main data platform, users can explore their data across modalities using additional web-based tools.

### Loading Raw Files Into the Relational Database

In a typical implementation of a workflow in this framework, raw data is imported into the LORIS data system and stored or linked in its relational tables. For the Ludmer Centre’s pilot implementation, data on 328 subjects from the Maternal Adversity, Vulnerability and Neurodevelopment study (MAVAN; O’Donnell et al., 2014) were processed and stored in LORIS. Data collected and stored on these subjects included questionnaires, demographic and phenotypic information and imaging scans.

Biosamples from each subject were collected, stored, and then processed by a third-party genotyping facility. The resulting IDAT files were run on the the *Methylation450k* pipeline and then transferred via a project-specific *DataProvider* to CBRAIN. This output was stored on CBRAIN as a large (CSV) matrix of 328 columns (samples) and 450,000 rows (probes) of Beta-values. This file was transferred to the LORIS server via SFTP and its contents were loaded into LORIS along with the Illumina annotation records. The *Genomic Uploader* module in LORIS was used to do this, creating a bio-sample record that associated over 450,000 values with each subject in LORIS. As a result, more than 147,600,000 values were stored in the *genomic_cpg* table.

In parallel, SNP data from these processed biosamples were transferred in the form of PLINK files (.PED and .MAP format) from a private FTP site to the LORIS server. These data points were transformed via PLINK commands and loaded into the LORIS database. SNP annotations were taken from the *dbSNP^25^* resource database to build filters on individual SNP values in the *Genomic Browser*.

### Selection, Filtering and Visualization Within the LORIS Data Platform

With several modalities of data for the population now stored in LORIS, the *Genomic Browser* and *Genomic Viewer* were used to select and filter variables of interest across data types. With the *DatasetBuilder*, new datasets were then defined by joining across other modalities, and can serve as input for later processing tasks to be launched on the HPC grid via CBRAIN.

#### Genomic Browser

**Figure 4:**
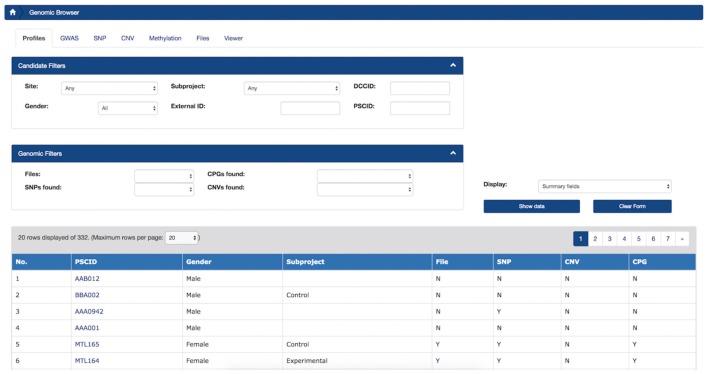
LORIS Genomic Browser: Profiles tab. Filter applied to search for subjects based on Site, Gender, Subproject, External ID and the availability of genomic data. In the table, detailed subject data can be accessed by clicking on the link that appears on each item.

**Figure 5:**
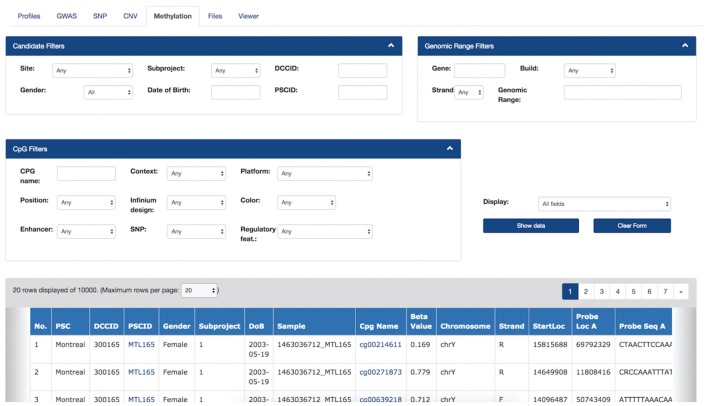
Filters and Methylation Beta-values in the Genomic Browser. Filters are applied on subject information, genomic range and the probe’s annotations. The filtered data view can be downloaded as a CSV file. Hyperlinks on each “CpG Name” column cell will bring the user to the online UCSC genome browser, which provides detailed information about a given CpG from the most recent human genome build version.

For researchers, a key feature is linking cross-modal data using a simple interface with querying, visualization, and search capabilities. The *Genomic Browser* (Figures 4, 5) enabled filtering values by their annotations, such that genomic data was uploaded and imported into LORIS, and then analyzed and visualized.

#### Genomic Viewer

**Figure 6:**
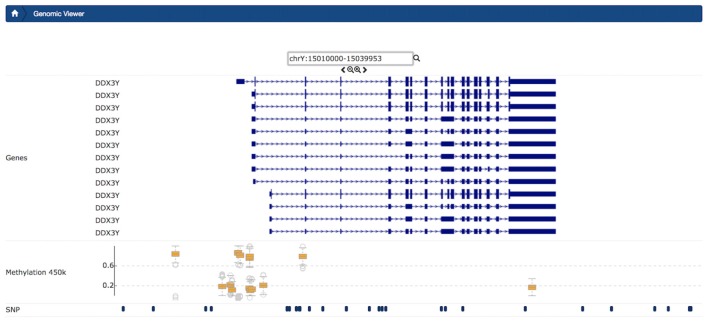
Example Genomic Viewer shows the context for single-nucleotide polymorphisms (SNPs) and CpGs in a small region of CpGs. Visualized context includes features from external sources, for chromosome Y from position 15010000 to 15039953. The upper section of the visualization plot presents the transcripts of gene DDX3Y with 5′UTR, as well as exons and transcription direction dynamically queried from the UCSC Genome browser. In the middle track, box plot distributions show Beta-values for each CpG. In the lowest track, in this view, users can view SNP and CpG positions stored in LORIS.

For each subject’s methylation data, the *Genomic Viewer* tab (Figure 6) displayed detailed genomic information. In this tab, users could view aggregated CpG Beta-value distributions visually aligned with SNP data alongside salient gene features for a given range on the genome. This module complemented more sophisticated and domain-specific tools by providing an intuitive web-accessible exploration utility directly within the context of the database, aligning all data points for all subjects of interest on the genome. The ability to “zoom in” on the genome, to better contextualize the measurement of interest, facilitates understanding of the data within a unified platform. Additional “tracks” from the UCSC Genome Browser are dynamically displayed to provide context for displayed CpGs and SNPs.

#### DatasetBuilder

**Figure 7:**
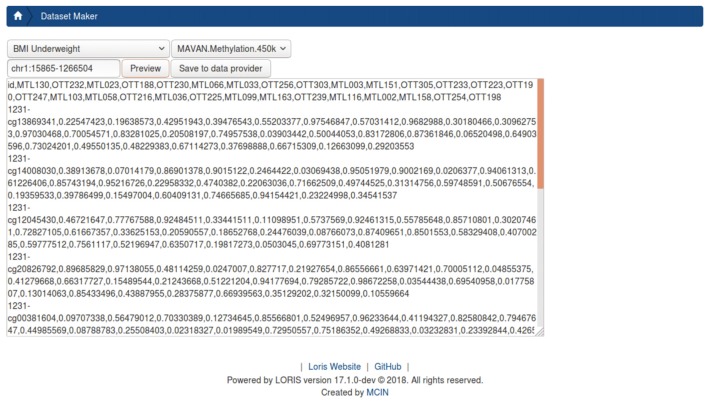
Prototype DatasetBuilder module. The preview panel displays all records returned from jointly querying the database, using the “BMI underweight” pre-built query stored in the data querying tool (DQT) module. This is joined with all subject-samples on which CpGs were found on chromosome 1 between position 15865 and 1266504 from the Methylation450k dataset Beta-values.

Once genomic data have been filtered and collated, the *DatasetBuilder* (Figure 7) allows users to aggregate phenotypic, imaging, and other modalities of data for a range of variables across all subjects. A custom dataset can be filtered for specific genomic regions of interest. An intuitive interface design leads users through a process of selecting a genomic fileset, targeting ranges of interest on the genome, and then cross-joining these results by subject ID based on a pre-constructed query across other modalities. The results are saved on the *DataProvider* directory file structure, ensuring that they are available to CBRAIN.

### CBRAIN Execution of Containerized Tools

Several pipelines have been made available through CBRAIN for the MAVAN study, such as the *Methylation450K* and *imputePrepSanger*^26^ *PCEV*, all described and running in containers. Once installed on CBRAIN and freely available to the community, users can launch these pipelines for their project easily on a number of available HPC resources without any need for additional installation or setup.

The above-mentioned pipelines are spawned as tasks on HPC clusters, where they process data accessed via the *DataProvider*. The output formats described for the pipeline are predefined and remain consistent. These pipelines can be updated on CBRAIN with new versions which may include updates to data format definitions.

Recent work on both LORIS and CBRAIN allows for task creation to spawn processes on CBRAIN where each instance is logged in the LORIS database. Provided an existing tool is registered on CBRAIN and the *DataProvider* is set up, LORIS can register files on CBRAIN and launch an analysis process on them using CBRAIN’s RESTful API. Once files are registered on a *DataProvider*, they are recognized by CBRAIN, and transferred to HPC units without any user intervention.

### Applications of Additional Pipelines for Derived Data

After pre-processing datasets using containerized pipelines on CBRAIN, additional pipelines can be executed on selected datasets from LORIS in a similar manner. Populations and fields of interest are identified, the datasets are sent to CBRAIN, and then a particular container-defined pipeline can be launched. All of these steps can be customized in order to enable execution from the LORIS front-end. Derived datasets from pipeline runs can be generated and returned to LORIS in a similar manner. As mentioned above, users also have the flexibility to re-run desired pipelines with altered parameters in subsequent stages to compare the results within or between pipelines.

**Figure 8:**
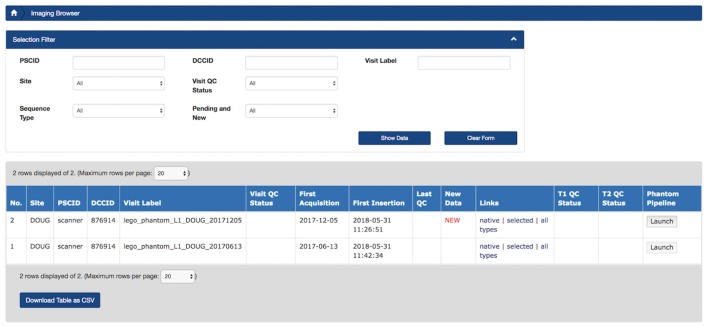
Prototype of LORIS Imaging Browser with PhantomPipeline processing launch capability using a single button. A user can click on the “Launch” button, under the “PhantomPipeline”column to initiate transfer of the scan dataset to CBRAIN to begin execution of the task.

**Figure 9:**
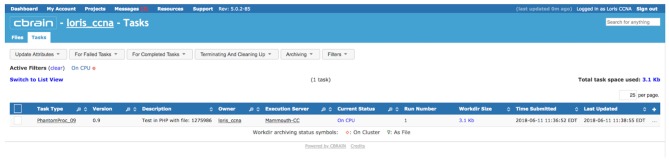
View of task (PhantomPipeline) running on CBRAIN web portal, launched from LORIS Imaging Browser module in Figure 8. The task was launched automatically through CBRAIN’s application programming interface (API), but can also be viewed and monitored interactively this way.

Beyond the Ludmer Centre pilot project, applications of this model have been tested on neuroimaging datasets for the Canadian Consortium for Neuroimaging in Aging (CCNA, Mohaddes et al., 2018, this issue). Derived data from MRI lego phantom processing (Fonov et al., 2010) plays a key role in identifying and correcting scanner distortion on scans collected across the CCNA network. LORIS’ Imaging Browser (Figure 8) is being customized to support automatic launching of the *PhantomPipeline* (Fonov et al., 2010) for execution through CBRAIN (Figure 9).

A key advantage of this framework is reproducibility of results, facilitated by detailed provenance capture (logs and parameter definitions from each processing step), as well as container technology (Merkel, 2014) to encapsulate the software environment used for processing and enabling rapid re-deployment.

## Discussion

This article focuses on the integration of “*omics”* data with phenotypic data to describe a novel framework for multimodal workflows. One of the key advantages of this model is the variety of functions and tasks covered within a single access-controlled system, such as enhanced monitoring of tasks, provenance tracking and storage of results and visualization features. Improving setup time for installation and re-deployment of containerized pipelines, and abstraction of HPC execution complexities also serve to remove constraints on researchers embarking on the computational learning curve. That being said, the most important aspect of a generalizable framework is to streamline processing and analysis through automation and standardization. Our use-case concretely exemplifies those steps through: (i) containerizing the *Methylation450K* and *ImputePrepSanger* pipelines in CBRAIN; (ii) launching and relaunching analysis from LORIS using APIs; and (iii) returning results to the *Genomic Browser* module in a structured manner.

Another important element to consider is that in many research environments, workflows are typically processed without the benefits of automated tools or computational infrastructure leading to inefficiencies, disorganization and with time, unmaintained datasets (Siebra et al., 2012). This has become increasingly evident in collaborations that require data sharing, scaling, or re-analysis. As such, we have leveraged established infrastructure to remove or abstract the complexities of data management from the end-user. This is of particular importance given that not all researchers have the time, interest, or expertise to manage the technical aspects of pipeline design and implementation of HPC execution on large datasets. The benefits of organized and curated datasets (Van Horn and Toga, 2009; Kanwal et al., 2017; Nichols et al., 2017; NIH Data Sharing Policy) have been reinforced through the generalizable framework described in this article. While it is true that there are a plethora of software tools and platforms that seek to reduce the technical burden on researchers, not all of them incorporate the full array of best practices necessary for ensuring reproducibility and accuracy in scientific analysis. Our main focus has been to leverage those missing pieces, namely standardization, provenance capabilities, interoperability between systems (such as HPCs) and enhance them with multimodal capabilities and effective visualization of data.

The ability to cross-link -*omic* output with phenotypic and imaging datasets is becoming an increasingly important factor in analysis. Cross-modal linking enables centralized sharing of richer study datasets within a network of investigators, establishing common dataset versions among researchers, and reducing the diffusion of multiple versions of similar datasets. In environments where computational infrastructure is lacking, a great deal of time is typically spent manually organizing datasets in spreadsheets and linking multi-modal data (Calabria et al., 2015). The *Genomic Browser* we describe provides an at-a-glance view of the available data for each participant within LORIS. It also provides a transparent and reproducible capability for visualizing genomic data by enabling filtering and querying across all available data types on shared properties and specific genomic regions around genes of interest. All of these features are graphically displayable on the *Genomic Viewer*. At the same time, the *DatasetBuilder* assembles multimodal datasets to run on processing pipelines in an automated and reproducible manner to significantly improve reliability of data outputs and traceability of targeted datasets. Looking towards a broader use-case, integration of genetics with other data types in a single platform can facilitate validation of genotypic vs. phenotypic characteristics. Basic validations of reported/phenotypic sex compared to genomic sex in a population and comparing reported ethnicity to genomic population markers are common examples. Such functions, which consider participant-specific phenotypes, allow for multi-level data integration, which are lacking in many existing online informatic resources e.g., GTEx.

Pursuant to utilizing an established data management platform, the benefits of standardization are an important topic and become evident in the execution of pipelines. A key example is how standardizing software installation through container technology reduces potential errors in the configuration and deployment of such pipelines. At the same time, it enhances portability to other platforms, irrespective of the operating systems (Roure et al., 2011; Cito et al., 2016; Sochat et al., 2017), while ensuring the pipelines are consistently executed across networks and research applications. This standardized execution and storage model can be generalized and scaled to larger, more complex workflows and multimodal data types ranging from other kinds of biological “*omics*” data (transcriptomics, proteomics, blood sugar, anthropometry) to behavioral, imaging and electrophysiological data, among others (Zhao et al., 2008). Beyond the example of the *Methylation450k* pipeline, this framework can be used to run any other processing task supported in CBRAIN, yet launched through LORIS. Currently, development is underway to use Galaxy to design additional workflows, and further optimize the *PCEV*^27^ pipeline. This pipeline is however only one amongst many other analysis methods that can be used in imaging genetics (Vilor-Tejedor et al., 2018).

Provenance also remains an important issue in any kind of analysis, especially in a multi-modal and multi-software environment, such as the generalizable workflow proposed in this article. To ensure complete accessibility of provenance information:

- task log details from CBRAIN’s internal records are communicated to LORIS with each set of returned results and made queryable via the LORIS front-end.
- standard file formats (e.g., JSON, XML, TSV) are used for the re-insertion process for derived data, as well as metadata to facilitate integration into LORIS with minimal interface development.
- quality control results are stored alongside raw and processed outputs which improves usability.
- increasing adoption of *Boutiques* descriptors (Glatard et al., 2018) as a framework for sharing and defining task creation on HPC resources will support standardization and transparency in neuroinformatic analyses.

The ultimate aim is to produce results and maintain provenance information that is compatible with emerging neuroimaging standards (e.g., the NIDM, Keator et al., 2016).

Interoperability between systems and datasets has become a requirement for sharing and collaboration in numerous fields involving many complex analytics, such as machine learning algorithms which are a rising interest in the field of imaging genetics. Making use of APIs that can seamlessly operate from one environment to another is a key consideration in our model. Linking to other systems to share data, or simply for reference pointers (e.g., links to the UCSC Genome Browser), is an important step in data harmonization (Zaveri, 2017). Developing APIs that are streamlined across platforms and easily fulfill community standards and workflow requirements provides an important asset for interoperability in large-scale consortia and open data initiatives (Poline et al., 2012; Poldrack et al., 2013; Van Horn and Toga, 2014; Craddock et al., 2016; Das et al., 2017).

One key advantage of this infrastructure is “Privacy by Design” which uses several mechanisms from acquisition to dissemination to ensure privacy, such as anonymous identifiers that link epigenetic data to a subject record, encryption methods to secure data transfers, specific anonymization techniques and other best practices (Cavoukian, 2009). This method largely removes the need to store personally identifying information (e.g., research participants and patient names) further mitigating the risk of re-identification. This facilitates sharing of other available data elements with a detailed provenance history when publishing analyses of genomic data through LORIS, where permissible, and in compliance with ethical regulations. Rendering these datasets non-identifiable is an active research area, giving rise to masking algorithms, which may be of interest to data-sharing initiatives.

Another major challenge in analysis is reproducibility. This becomes particularly evident in workflows that span different domains such as imaging and genetics (Nekrutenko and Taylor, 2012). In its process design and technical implementation, this generalizable framework aims to adhere to the *FAIR* (Wilkinson et al., 2016*)* data principles. In our workflow, inputs and outputs of each processing task are available to platform members alongside provenance information from container descriptions and pipeline execution logs, and each step of the workflow can be re-run locally or on other systems. Using the open-source constituent tools of this workflow, capturing the same outputs in the same manner from a reproduction of this workflow provides a powerful means to directly compare each aspect of an analysis that has been re-run.

Through the development of this combined framework and across several infrastructure initiatives, best practices have emerged. These have been articulated in Appendices 1 and 2 as guidelines summarizing both the principles and practical recommendations for implementations of this framework.

**Future extensions** of this infrastructure, based on user feedback, will add richer features and more seamless automation at several stages. As a result, a number of features will be developed and improved:

- Streamlining the data-loading processes in LORIS via the release of open-source tools will facilitate easier adoption of this framework for other “*omics”* workflows.
- Integrating formats from other platforms will expand the scope of this technology.
- Address scaling challenges through increasing use of NoSQL schema-less databasing to flexibly handle increasing volumes of genomic data and its significant variability across data types and structures.
- *Boutiques* descriptors for CBRAIN to generalize LORIS task-launching capabilities and ease the development burden of deploying new pipelines.
- A well-defined API using the OpenAPI^28^ standard, registered on SmartAPI^29^, to facilitate the creation of specialized tools to interact with LORIS programmatically.
- Interoperability with data discovery platforms like *DataLad*^30^, to support querying, packaging and return of LORIS-hosted data into BIDS^31^-formatted data objects. Adding enhanced support for API endpoints will support these operations.
- Encapsulating the *Genomic Viewer* into a Javascript module would help portability across platforms.

While these components will fulfill the vision for a fully robust feature-set in LORIS and CBRAIN, further developments, documentation, unit tests and integration tests will be important to include beyond the prototyping stage, to ensure the resulting combined framework does not amass technical debt for future workflows.

## Conclusion

The goal of this article is to present a novel framework that can facilitate brain research discovery by reducing human error through the automation of analysis pipelines and seamless linking of multimodal data workflows. The described framework for *“omics”* workflows integrates multi-modal data support in a mature databasing system with analysis on HPC platforms, with a wide array of capabilities including provenance tracking, a well-defined processing environment, visualization, querying and links with other existing genomics databases. Ultimately, this framework aims to create an optimally user-friendly experience to allow researchers to focus on scientific aims rather than the obstacles that otherwise occur with complex data handling.

## Author Contributions

SD, XLB, CR, MF, NB and CG contributed to the conception and design of the generalized workflow and wrote sections of the manuscript. FC-D, CM, PR, SB, VF and CZ wrote sections of the manuscript. All authors contributed to manuscript revision, read and approved the submitted version.

## Conflict of Interest Statement

The authors declare that the research was conducted in the absence of any commercial or financial relationships that could be construed as a potential conflict of interest.

## Footnotes

^1^Such as transcriptomics, proteomics, blood sugar, anthropometry, etc.

^2^http://ludmercenter.ca

^3^http://www.loris.ca

^4^http://mcin-cnim.ca/technology/cbrain/

^5^https://github.com/GreenwoodLab/methylation450KPipeline

^6^http://jupyter.org/

^7^Representational State Transfer (REST) is an software architecture style compliant with Hypertext Transfer Protocol (RFC 2616) where each url is a resource that can be interact with using verbs (GET, PUT, POST, DELETE, etc.).

^8^www.computecanada.ca

^9^A task is an instance of a tool running on CBRAIN where a tool is any piece of software that take inputs and generates outputs installed on CBRAIN.

^10^Docker containers are units of processing where tool versions, an environment (OS), and sequences of operations can be reproduced on any system.

^11^Singularity is another container technology that has been privileged over Docker on HPC units served by CBRAIN.

^12^Container description files are versioned text files that contain the recipe to (re)build a given container image; they present themselves as a sequence of shell commands.

^13^https://hub.docker.com/r/eauforest/imputeprepsanger/

^14^https://www.sanger.ac.uk/science/tools/sanger-imputation-service

^15^Variant Call Format. A specification to encode genetic variations in a text file.

^16^https://github.com/GreenwoodLab/pcev_pipelineCBRAIN

^17^https://editor.swagger.io/

^18^https://github.com/aces/cbrain/blob/master/BrainPortal/public/swagger/cbrain-4.5.1-swagger.yaml

^19^https://genome.ucsc.edu/

^20^https://reactjs.org/

^21^https://d3js.org/

^22^Beta-values represent levels of DNA methylation at a given probe (CpG) and range from 0 to 1, representing 0%–100% DNA methylation at a given site.

^23^https://github.com/aces/Loris/blob/master/modules/genomic_browser/tools/HumanMethylation450k_annotations_to_sql.py

^24^Category of functions that split a problem into parallelizable parts so it can run on multiple threads and/or distributed computers.

^25^https://www.ncbi.nlm.nih.gov/projects/SNP/

^26^https://hub.docker.com/r/eauforest/imputeprepsanger

^27^https://github.com/GreenwoodLab/pcev_pipelineCBRAIN

^28^https://github.com/OAI/OpenAPI-Specification

^29^https://smart-api.info/

^30^https://github.com/DataLad/DataLad

^31^https://github.com/INCF/BIDS; BIDS is an emerging neuroimaging data standard for describing and sharing data and metadata.

## Appendix

**Preface to Appendices:** The authors have developed and recommend community-supported best practices for adhering to *FAIR* principles in both the development of infrastructure and its implementation in practice.

### Appendix 1

Best Practices checklist for technology design and development for *FAIR Multimodal* Framework integration:

***Findable***:

- Recognized by SmartAPI registry
- Use DOI for datasets and link them to LORIS API endpoint
- Softwares openly available via public repositories, and free (as in Freedom^32^)
- Connect datasets using technologies like DataLad
- Publish and cite others

***Accessible***:

- Must be governed by the study and subject consent
- Ensure protection of https but no institution-restricting firewall (hospitals)
- Sustainable infrastructure plan to backup, support and maintain the server and storage. This includes software security upgrades to keep data safe as well as accessible

***Interoperable***:

- API endpoints for datasets + version (GitAnnex/DataLad)
- Converters for BIDS—import/export of datasets
- Provide guidance where possible on use with other standards such as NIDM

***Reusable***:

- Container technologies
- Boutiques descriptors

- Software pipelines coded in a way that it can be reused
- Reuse of analysis results: ensure execution parameters and Provenance information is readily accessible *and* shareable with the data (so they can be exported together)

### Appendix 2

Implementation Guidelines for *FAIR Multimodal* Workflow integration:

- Store raw data at an early stage; this facilitates the linkage between subjects and the provenance “trail”
- Use a centralized database system with user authentication for full auditing and data organization
- Describe entities, agents and activities with *PROV^33^* family vocabulary.
- Use https and ssh as protocols of communication
- Use a network with known geographical location of nodes (HPC, Storage, Hubs)
- Design pipelines with checkpoints where the state of the data can be reused with alternative paths (forks)
- Use container technology to describe the execution environment setup
- Publish software (web-apps, pipelines, containers) in a public hub with appropriate licenses—ideally free (as in freedom)
- Document software for users but also future contributors, including “contribution guidances”
- Describe pipeline requirements: space, CPUs, nodes, formats (Kanwal et al., 2017)
- Cite all used software and datasets
- Publish datasets on sustainable platforms
- Describe your datasets with common terms and standard community-supported formats (JSON-LD, RDF)
